# Correction of human phospholamban R14del mutation associated with cardiomyopathy using targeted nucleases and combination therapy

**DOI:** 10.1038/ncomms7955

**Published:** 2015-04-29

**Authors:** Ioannis Karakikes, Francesca Stillitano, Mathieu Nonnenmacher, Christos Tzimas, Despina Sanoudou, Vittavat Termglinchan, Chi-Wing Kong, Stephanie Rushing, Jens Hansen, Delaine Ceholski, Fotis Kolokathis, Dimitrios Kremastinos, Alexandros Katoulis, Lihuan Ren, Ninette Cohen, Johannes M.I.H. Gho, Dimitrios Tsiapras, Aryan Vink, Joseph C. Wu, Folkert W. Asselbergs, Ronald A. Li, Jean-Sebastien Hulot, Evangelia G. Kranias, Roger J. Hajjar

**Affiliations:** 1 Cardiovascular Research Center, Icahn School of Medicine at Mount Sinai, New York, New York 10029, USA; 2 Department of Medicine, Division of Cardiovascular Medicine, Stanford University School of Medicine, Stanford, California 94305, USA; 3 Stanford Cardiovascular Institute, Stanford University School of Medicine, Stanford, California 94305, USA; 4 Molecular Biology, Biomedical Research Foundation of the Academy of Athens, Athens, Greece; 5 Pharmacology Department, University of Athens, Athens, Greece; 6 Stem Cell & Regenerative Medicine Consortium, Department of Physiology, LKS Faculty of Medicine, University of Hong Kong, Hong Kong, China; 7 Department of Pharmacology and Systems Therapeutics, Systems Biology Center New York, Icahn School of Medicine at Mount Sinai, New York, New York 10029, USA; 8 Attikon Hospital, University of Athens, Athens 12462, Greece; 9 Department of Genetics and Genomic Sciences, Icahn School of Medicine at Mount Sinai, New York, New York 10029, USA; 10 Department of Cardiology, Division Heart and Lungs, University Medical Centre Utrecht, PO Box 85500, 3508 GA, Utrecht, The Netherlands; 11 Ona ssis Cardiac Surgery Center, Athens, Greece; 12 Department of Pathology, Division Laboratories and Pharmacy, University Medical Center Utrecht, PO Box 85500, 3508 GA, Utrecht, The Netherlands; 13 Durrer Center for Cardiogenetic Research, ICIN-Netherlands Heart Institute, Utrecht, The Netherlands; 14 Institute of Cardiovascular Science, faculty of Population Health Sciences, University College London, London WC1E 6BT, UK; 15 Sorbonne Universités, UPMC Univ Paris 06, INSERM, UMR_S 1166 ICAN, F-75005 Paris, France; 16 Department of Pharmacology, University of Cincinnati, Cincinnati, Ohio 45267-0575, USA

## Abstract

A number of genetic mutations is associated with cardiomyopathies. A mutation in the coding region of the phospholamban (*PLN*) gene (R14del) is identified in families with hereditary heart failure. Heterozygous patients exhibit left ventricular dilation and ventricular arrhythmias. Here we generate induced pluripotent stem cells (iPSCs) from a patient harbouring the *PLN* R14del mutation and differentiate them into cardiomyocytes (iPSC-CMs). We find that the *PLN* R14del mutation induces Ca^2+^ handling abnormalities, electrical instability, abnormal cytoplasmic distribution of PLN protein and increases expression of molecular markers of cardiac hypertrophy in iPSC-CMs. Gene correction using transcription activator-like effector nucleases (TALENs) ameliorates the R14del-associated disease phenotypes in iPSC-CMs. In addition, we show that knocking down the endogenous *PLN* and simultaneously expressing a codon-optimized *PLN* gene reverses the disease phenotype *in vitro*. Our findings offer novel strategies for targeting the pathogenic mutations associated with cardiomyopathies.

## Abstract (web summary)

Phospholamban (PLN) is a regulator of heart contractility. Here the authors show that cardiomyocytes derived from induced pluripotent stem cells of a cardiomyopathy patient with mutant PLN exhibit functional defects consistent with the disease, and that this mutation can be functionally corrected by genome editing and gene therapy.

## Graphical abstract

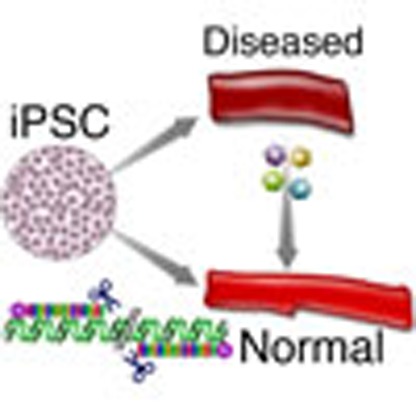

Dilated cardiomyopathy (DCM) is a leading cause of heart failure characterized by biventricular dilatation and progressive cardiac dysfunction and is often accompanied by ventricular arrhythmias^1^. Recent studies have unravelled the genetic basis of many forms of DCM, leading to the identification of pathogenic DCM-causing mutations in a diverse set of myocardial genes^2^, including phospholamban (also known as *PLN*)^3^,^4^,^5^,^6^. *PLN* is a regulatory protein that controls Ca^2+^ cycling in cardiomyocytes (CMs)^7^. *PLN* inhibits the cardiac isoform of the sarcoplasmic reticulum (SR) Ca^2+^ ATPase (SERCA2a) in cardiac myocytes thereby reducing influx of calcium into the SR. PLN is phosphorylated at Ser16 by PKA or Thr17 by calcium/calmodulin-dependent protein kinase II (CaMKII) or protein kinase B (Akt). When phosphorylated at either or both of these sites, the inhibition of SERCA2a is alleviated and calcium flux into the SR increases. Several mutations in *PLN* have been identified in patients and linked to hereditary DCM. The precise cardiac pathology of the different PLN mutations varies but most patients present with cardiac dilatation, hypertrophy, decreased ejection fraction and ventricular arrhythmias. A deletion of amino acid arginine 14 in the coding region of the *PLN* gene (R14del) was first discovered in a large Greek family with hereditary DCM^5^ and DCM patients heterozygous for the R14del mutation exhibit left ventricular dilation, contractile dysfunction and episodic ventricular arrhythmias, with overt heart failure by middle age in some cases^5^,^8^,^9^. Although the molecular mechanism underlying the pathogenesis of R14del-induced DCM remains unclear, experimental evidence in a mouse model has suggested a link between *PLN* R14del mutation and the impairment of cardiac Ca^2+^ cycling, an effect that may be related to destabilization of the pentameric structure of *PLN* and SERCA2a super inhibition^5^. However, there was no effect on SERCA2a activity when the R14del *PLN* was expressed in the *PLN*-null background mice^10^. Interestingly, the R14 deletion of *PLN* has been associated with both mild and severe forms of cardiomyopathy with variable degrees of arrhythmias 5,8,11. More recently, a study found that *PLN* R14del mutation carriers are at high risk for malignant ventricular arrhythmias and end-stage heart failure, with left ventricular ejection fraction <45% and sustained or nonsustained ventricular tachycardia as independent risk factors^12^.

The ability to generate patient-specific human induced pluripotent stem cells (iPSCs) offers new opportunities to study the underlying mechanisms of DCM pathogenesis that can eventually enable the development of personalized therapies. Indeed, inherited cardiovascular diseases have been modelled in iPSCs, including cardiac arrhythmia syndromes^13^,^14^,^15^,^16^,^17^,^18^,^19^,^20^,^21^,^22^, arrhythmogenic right ventricular cardiomyopathy^23^, familial hypertrophic cardiomyopathy^24^, Friedreich's ataxia^25^ and DCM^26^. The patient-specific iPSC-derived CMs recapitulate the pathogenic disease phenotype and could provide opportunities for novel insights into cardiac pathophysiology. The derived CMs develop specific features such as abnormal contractile function, dys-regulated calcium movements, altered electrical activities and an increase in hypertrophic markers and can serve as a platform to model the disease. When causative genetic mutations are present, genome engineering using designer nucleases can be used to correct the mutations. Three different types of molecular scissors have been developed that can be designed to cut at specific sites: (1) zinc finger nucleases, (2)transcription activator-like effector nucleases (TALENs) and (3) clustered regularly inter-spaced short palindromic repeats (CRISPR)^27^. Each of these designer nucleases have unique features, and their capability to target specific DNA sequences make them a very useful tool for manipulating iPS cells.

In this study, we derived iPSC-CMs from a patient with cardiomyopathy associated with a *PLN* R14del mutation to better understand the link between mutant R14del and cardiac pathophysiology. The patient-specific CMs displayed Ca^2+^ handling abnormalities, electrical instability, abnormal cytoplasmic distribution of PLN protein and an increased expression of molecular markers associated with cardiac hypertrophy. Using TALEN-mediated gene editing, we corrected the *PLN* mutation in iPSCs and the derived CMs display a normal phenotype. Furthermore, the abnormal phenotype in iPSC-derived CMs carrying the R14del mutation was reversed by a combinatorial adeno-assisted vector (AAV)-mediated gene therapy approach to knockdown the endogenous *PLN* while expressing a codon-optimized human *PLN*.

## Results

### Derivation of iPSCs from a patient with PLN R14del mutation

We obtained a skin biopsy from a female patient carrying a heterozygous mutation in the *PLN* gene (c.40_42delAGA; p.Arg14del) who belongs to a large family with a long clinical history of hereditary DCM^5^ (VI 32 in Fig. 1 of ref. 5). The patient presented with mild left ventricular systolic dysfunction, ventricular chamber dilatation and frequent ventricular arrhythmias. The typical sustained ventricular arrhythmias and left ventricular dysfunction characteristic of these patients are depicted in Fig. 1a and Supplementary Movie 1. Histopathological examination of myocardial sections obtained from explanted hearts of patients carrying the *PLN*-R14del mutation that derived from a different founder showed an abnormal cytoplasmic distribution and perinuclear accumulation of PLN (Fig. 1b), suggesting that the R14del mutation is altering the intracellular localization of the PLN. We generated three individual iPSC clones (designated L1, L2 and L3) from patient-specific dermal fibroblasts carrying the R14del mutation (hereafter referred to as iPSC-R14del) using the synthetic modified messenger RNA (mRNA) reprogramming system^28^ (Supplementary Fig. 1a). The heterozygous mutation was verified by sequencing of the *PLN* coding region in both the dermal fibroblasts and the fibroblast-derived iPSC-R14del clones (Supplementary Fig. 1b). Multiple assays were used to confirm the successful reprogramming of the fibroblasts to iPSCs. Immunostaining analysis demonstrated that the iPSC-R14del lines expressed the pluripotency markers *NANOG, OCT-3/4, SOX-2* and *SSEA-4* (Fig.1c and Supplementary Fig. 1c). We also assessed pluripotency and tri-lineage differentiation potential using real-time quantitative PCR assays. We compared the gene expression profiles of the iPSC clones to a reference set of embryonic stem cells (ESCs) and iPSC lines using a bioinformatic scorecard approach^29^. The results showed that the R14del-iPSC clones were transcriptional similar to the reference set and differentiated into all three embryonic germ layers *in vitro* (Supplementary Fig. 1d). Moreover, digital karyotyping analysis showed that all the iPSC clones were euploid and no chromosomal aberrations were observed (Supplementary Fig. 1e).

### Functional characteristics of iPS-CM with *PLN* R14del mutation

We differentiated the iPSCs-R14del from the three patient-specific clones (L1, L2 and L3) into CMs using a directed differentiation method that yields a high percentage of ventricular-like CMs^30^. After 15 days of differentiation, we observed a widespread spontaneous beating activity of the iPSC-R14del differentiation cultures (Supplementary Movie 2), which is a characteristic of functional CMs (hereafter referred to as R14del-CMs). Cultures of differentiated cells stained positive for cardiac Troponin T, a marker of terminally differentiated CMs. Also, we confirmed the expression of PLN in R14del-CMs (Supplementary Fig. 2). As PLN plays a central role in modulating CM calcium (Ca^2+^) homeostasis by regulating the activity of SERCA2a^31^, we next determined whether the R14del mutation is affecting Ca^2+^ handling in iPSC-derived CMs. We recorded the basal intracellular Ca^2+^ ([Ca^2+^]_i_) transients upon electrical stimulation of R14del-CMs and WT-CMs loaded with fura-2AM, a Ca^2+^ indicator. We observed a progressive dysregulation of Ca^2+^ cycling in differentiated R14del-CMs, which exhibited an arrhythmic Ca^2+^ cycling profile (Fig. 1d,e), characterized by a higher spontaneous beating rate and frequent episodes of irregular Ca^2+^ waves when compared with control CMs (Fig.1f). Taken together, these data demonstrated impairment in Ca^2+^ handling properties of iPSC-derived CMs characterized by arrhythmias, suggesting a critical role of abnormal Ca^2+^ handling in the pathogenesis of DCM induced by the R14del mutation of *PLN*.

### Genomic editing of iPSCs with PLN R14del mutation

Given that epigenetic and transcriptional variation is common among human pluripotent cell lines^32^, we sought to determine whether the Ca^2+^ handling abnormalities observed in R14del-CM were specifically induced by the R14del mutation. We developed a TALEN vector pair^33^ designed to introduce a double-strand break adjacent to the R14del mutation of the *PLN* and validated its efficiency by the surveyor assay^34^ (Supplementary Fig. 3). Patient-derived iPSCs were co-transfected with a PLN-specific TALEN pair together with the gene correction matrix (Fig. 2a). We obtained two isogenic iPSC clones, designated L2GC1 and L2GC2, which differ only in the R14del mutation with the parent clone L2. The corrected clones were expanded and the correction of the R14del mutation was confirmed by Sanger sequencing (Fig. 2b and Supplementary Fig. 4). Similarly to the parental line, the TALEN-edited isogenic iPSCs clones were characterized and confirmed to be pluripotent and karyotypically normal (Supplementary Fig. 5). We differentiated the corrected clones into CMs, and determined that the targeted gene correction did not alter the gene expression level of *PLN* and appropriate expression of the ‘TALEN-corrected' allele was confirmed in the isogenic CMs of both clones by pyrosequencing (Fig. 2c). In addition, we performed whole-exome sequencing and bioinformatics analyses to assess off-target mutagenesis. Consistent with recent reports^35^,^36^,^37^, exome sequencing of the parental and TALEN-corrected iPSCs showed that off-target mutations attributable to the TALENs were very rare (Supplementary Table 1). Next we sought to determine whether the genetic correction of the R14del mutation resulted in functional phenotypic correction. We observed that TALEN-corrected CMs displayed a normal Ca^2+^ cycling phenotype (Fig. 3a–c), and a significantly lower resting diastolic Ca^2+^ levels compared to CMs derived from the the parent line (Supplementary Fig. 6). Previously, it was shown that Ca^2+^-induced Ca^2+^ release machinery is functional in iPSC-derived CMs^38^. Indeed, caffeine application elicited an instantaneous and rapid release of Ca^2+^ from the intracellular stores of CMs, resulting in high amplitude Ca^2+^ transients (Fig. 3d). Consistent with the hyperdynamic basal function of the R14del PLN^10^, the caffeine-induced Ca^2+^ amplitude was significantly higher in the R14del-CMs when compared with the TALEN-corrected CMs (Fig. 3e). Also, single-cell electrophysiological analysis demonstrated that R14del-CMs showed faster depolarization rate and significantly more depolarized maximum diastolic potential than the TALEN-corrected CMs (Fig. 4a,b and Table 1). In addition, the corrected CMs showed a regression of the hypertrophic phenotype, as evidenced by the reduced expression of the hypertrophic markers *ANF, BNP* and an increased *MYH6/MYH7* ratio (Fig. 4c). Interestingly, we observed that the subcellular distribution of the PLN protein in R14del-CMs was predominantly polarized at one side of the cytoplasm (Fig. 5), recapitulating the phenotype observed in the cardiac tissue sections of the R14del patients. In contrast, the corrected CMs displayed a homogeneous reticular distribution of PLN (Fig. 5). Taken together, these data strongly suggest that targeted gene correction of R14del PLN mutation is sufficient to fully restore a normal cardiac phenotype *ex vivo*.

### Correction of PLN R14del mutation by gene transfer in iPS-CMs

To achieve PLN correction by a combinatorial gene therapy approach, we engineered an AAV type 6 monocistronic vector allowing the knockdown of endogenous *PLN* by an intronic artificial microRNA (miR-PLN), together with overexpression of a microRNA (miRNA)-resistant, codon-optimized PLN (Fig. 6a). Self-complementary genomes were packaged into AAV6 capsids, which allow a high transduction efficiency of cardiac cells^39^. These vectors were used to infect R14del-CMs and allowed very high expression of wt PLN, together with ∼50% knockdown of endogenous *PLN* transcripts (Fig. 6b and Supplementary Fig. 7). Importantly, miR-mediated PLN knockdown was highly specific with no off-target effects, as assessed by RNA sequencing and bioinformatics analyses (Supplementary Fig. 8). Interestingly, AAV6-mediated overexpression of PLN induced a downregulation of endogenous PLN transcripts, suggestive of a negative feedback mechanism since the steady-state level of PLN in CMs is tightly regulated^40^ (Fig. 6b). To determine whether AAV6 vectors could rescue the Ca^2+^ abnormalities associated with the R14del mutation, we recorded the basal [Ca^2+^]_i_ transients in R14del-CMs. Seven days post AAV6 infection, we observed that AAV6-mediated PLN overexpression reduced the frequency of arrythmogenic episodes when compared with non-infected cells (Fig. 6c). The expression of the cardiac hypertrophy markers, *ANF* and *BNP*, was significantly increased following infection by control AAV6 particles, suggesting a general effect of AAV vectors on CM stress and homeostasis. However, infection by AAV6 encoding PLN resulted in a significant decrease of ANF expression down to a level comparable to that of normal iPSC-CMs (Fig. 6d). By contrast, BNP expression was not reduced down to the level observed in WT-CMs. Viral-mediated PLN expression also increased the *MYH6/MYH7* ratio of R14del-CM to a level exceeding that of normal CMs (Fig. 6e). Neither marker was significantly altered by the presence of PLN miRNA versus control miRNA, suggesting that overexpression of wild-type PLN alone is sufficient to improve CM functions without the suppression of mutant PLN. These findings suggest that AAV6-mediated overexpression of wild-type PLN was able to rescue the impaired calcium handling characteristics in R14del-CMs *in vitro.*

## Discussion

In this study, we demonstrate that CMs differentiated from mutant iPSCs, derived from a patient harbouring a deleterious R14del mutation in the *PLN* gene, display disease-specific irregular Ca^2+^ handling and abnormal cytoplasmic distribution of PLN protein. This irregularity in calcium cycling correlates well with the fatal arrhythmias observed in R14del patients. In addition, we observed an increase in the markers of cardiac hypertrophy and an abnormal cytoplasmic PLN protein distribution in CMs. Importantly, the abnormal PLN distribution was also observed in the myocardium of R14del patients. Using either a TALEN-mediated gene correction or a combinatorial gene therapy approach, we rescued the disease phenotype *in vitro*.

Genome engineering has been used to introduce disease genes in iPSCs to recapitulate disease phenotypes. In a recent study, the ion channel genes *KCNQ1* and *KCNH2* with dominant negative mutations causing Long QT syndrome (LQTS) type-1 and -2, respectively, were introduced into both ESCs and iPSCs using zinc finger nucleases^41^. The investigators then derived CMs, which displayed characteristics of LQTS phenotype including prolongation of the action potential duration, recapitulating the key pathophysiological features of LQTS types 1 and 2. In another study, Wang *et al.*^42^ modelled Barth syndrome, a mitochondrial cardiomyopathy disorder caused by mutation of the gene encoding Tafazzin (TAZ), with iPSCs obtained from patients affected by the disease or by Cas9-mediated genome editing to mutate TAZ in a control human line. Gene replacement and gene editing demonstrated that CMs exhibited metabolic, structural and functional abnormalities associated with TAZ mutations.

*In vivo* correction has also been performed in murine models. In a recent study by Jiang *et al.*^43^ the expression of a hypertrophic cardiomyopathy (HCM) causing mutation (Myh6 R403Q) was decreased in mice using RNA interference (RNAi). AAV-mediated Myh6-R403Q knockdown resulted in a reversal of myocardial fibrosis for ∼6 months^43^. In a separate study, Mearini *et al.*^44^ targeted a frame shift mutations in myosin-binding protein C3 (*Mybpc3*), encoding cardiac MyBP-C (cMyBP-C) associated with HCM. Administration of AAV9-Mybpc3 in 1-day-old transgenic mice prevented the development of cardiac hypertrophy and dysfunction for a period of 34 weeks^44^. In our study, we used a combinatorial gene therapy approach with knockdown of the endogenous mutant gene along with re-expression of the normal *PLN* gene. This gene therapy approach based on AAV9 delivery could be tested *in vivo* in an appropriate murine model of DCM carrying the R14del mutation.

Our findings may pave the way for the development of novel therapies of pathogenic mutations associated with inherited cardiomyopathies.

## Methods

### iPSC reprogramming

Dermal fibroblasts were obtained from a skin biopsy of a 42-year old volunteer following informed consent (Onassis Cardiac Surgery Center, Athens, Greece). Integration-free iPSC clones were generated from the patient's dermal fibroblasts carrying the R14del mutation using the synthetic modified mRNA reprogramming system^28^. Briefly, a reprogramming cocktail of five human mRNA reprogramming factors encoding OCT-3/4, SOX-2, c-MYC, KLF-4 and LIN-28 was used according to the manufacturer's protocol (Stemgent). Each mRNA factor was added at a concentration of 100 ng μl^−1^, resulting in an mRNA reprogramming cocktail with a stoichiometric ratio of 3:1:1:1:1 (for OCT-3/4, SOX-2, c-MYC, KLF-4 and LIN-28, respectively). The mRNA cocktail was delivered to the dermal fibroblasts cells daily using RNAiMAX Transfection Reagent (Invitrogen) diluted in Opti-MEM Medium (Invitrogen). Transfections were repeated at 24-h intervals for 15 days. Cell colonies were manually picked after ∼21 days and were propagated under feeder-independent conditions as described^45^. Briefly, iPSC lines were maintained in the mTeSR1 (Stem Cell Technologies) media on tissue culture plates coated with hESC-qualified Matrigel (BD Biosciences) in 5% CO_2_/5% O_2_/90% N_2_ environment at 37 °C.

### Differentiation of iPSC into CMs

The iPSC cells were differentiated into CMs using a direct differentiation method^46^. Cardiomyocyte differentiation was initiated in suspension cultures on ultra-low attachment dishes (Corning) in mTESR1 medium supplemented with BMP4 (10 ng ml^−1^) and Blebbistatin (5 μM) for 24 h. The medium was then replaced with the basal differentiation medium (StemPro34, 50 μg ml^−1^ ascorbic acid, 2 mM GlutaMAX-I) supplemented with BMP4 (10 ng ml^−1^) and Activin-A (25 ng ml^−1^) for 48 h (days 1–3) and then switched to basal differentiation medium for another 36 h (days 3–4.5). Finally, the cells were differentiated in basal differentiation medium supplemented with IWR-1 (2.5 μM) for 96 h (day 4.5–8.5). The differentiated CMs were maintained in basal differentiation media for up to 4 weeks. All cytokines were purchased from R&D systems. The small molecules were purchased from Sigma. All differentiation cultures were maintained in 5% CO_2_/air environment.

### Calcium transient analysis

At 30-days post differentiation, iPSC-derived CMs were dissociated, plated on matrigel-coated coverslips and cultured in basal medium for 72–96 h. The CMs were loaded with a fluorescent Ca^2+^ sensitive dye, Fura-2, and the ratios of fluorescence intensities (excited at 340 and 380 nm) were recorded using the Ionoptix system (Ionoptix). The electrically induced Ca^2+^ transients ([Ca^2+^]_i_) were triggered by pulses generated from a field stimulator (20 ms duration, 0.5 Hz). The Ca^2+^ traces were analysed using IonWizard software to calculate the irregularity of the timing (Beating rate), the percentage of irregular beating areas and the number of irregular waveforms.

### TALEN construction

TALEN genomic binding sites were designed using the TALEN Hit software (Cellectis Bioresearch). The TALEN genomic binding sites were 17 bp in length and the target sequence between the two binding sites was 17 bp in length (TALEN left: 3′-TTTATCAATTTCTGTCT-5′; TALEN right: 3′-TGATACAGATCAGCAAG-5′) separated by a 15 bp spacer. The homologous recombination (HR) matrix design, which spanned (nucleotides) nts 10,183 to 11,282 of the PLN gene, incorporated two silent mutations within each TALEN binding site, to prevent matrix DNA cleavage. The HR matrix was synthesized by Genescript and a PGK-Puromycin-2A-EGFP-SV40polyA selection cassette flanked by loxP sites was inserted in a unique BglII restriction site downstream of the PLN stop codon.

### Surveyor assay

The efficiency and specificity of the TALEN-induced modification of the PLN locus was validated by the SURVEYOR assay (Transgenomic) according the manufacturer's protocol. Briefly, TALENs were transfected into HEK-293T cells and 72 h later genomic DNA was extracted. The PLN locus was amplified by PCR (forward primer: 5′-GAGAGAGAGAGAGGGAGAGAGAC-3′ and reverse primer: 5′-GAAGTGAACTTGTTGGCAGTGCAG-3′) and the PCR amplification products were re-annealed, digested by the Surveyor nuclease and subjected to agarose gel electrophoresis to confirm TALEN-induced mutations.

### Isolation of targeted clonal cell populations

iPS-R14del cells were co-transfected with TALENs and HR matrix by electroporation^47^. On the day of the electroporation, semi-confluent cell cultures were dissociated into single cells with 0.025% trypsin for 5 min at 37 °C and 10^7^ cells were electroporated with a mix of 50 μg of the TALEN pair (25 μg of each plasmid) and 15 μg of the HR matrix in a 0.4 cm cuvette using the parameters 250 V and 500 μF. Following electroporation, the cells were re-plated at a low density and allowed to grow for about 5 days. Transfected clones were selected by puromycin (0.5 μg ml^−1^) for a period of 7 days. Puromycin-resistant colonies were picked up and transferred in duplicate into a matrigel-coated 48-well plate. Cells from one 48-well plate were used for DNA preparation ∼1 week later. *Bona fide* junctions between chromosome 6 and PLN matrix were screened by PCR using the forward primer 5′-GACCAGAAATATGCTATAGGAACTTAAC-3′ located on chromosome 6 upstream of the PLN matrix (PLN genomic positions 10,084–10,111) and the reverse primer 5′-CAGGGCTGCCTTGGAAA-3′ located in the PGK promoter of the selection cassette. To genotype the PLN allele present on the intact chromosome, a 746-nt fragment extending from PLN genomic positions 10,398 to 11,143 was amplified and digested with SapI endonuclease, which cuts only wild-type PLN, yielding two fragments of 464 and 279 nts (Supplementary Fig. 4).

### AAV vectors

Following selection of the best miRNA candidates, a SnaBI-NotI fragment containing most of the CMV promoter, the intronic miRNA and the EGFP sequence was excised from pSM155 vectors and inserted into the corresponding sites of self-complementary scAAV-EGFP^48^ to obtain scAAV-miRNA-EGFP vectors. To achieve simultaneous wild-type PLN overexpression, a codon-optimized miRNA-resistant version of PLN (PLNco) was synthesized and inserted between AgeI and NotI sites, in lieu of the EGFP, yielding scAAV-miR-PLNco vectors. scAAV vectors were pseudotyped into AAV6 capsids using the pDP-6 vector^49^ and purified by iodixanol gradient and dialysis. Viral titres were measured by real-time PCR using primers specific for the SV40 polyadenylation signal. IPS-CMs were infected at day 25 after the onset of differentiation using an multiplicity of infection (MOI) of 4 × 10^4^ viral genomes per cell.

### Artificial microRNA screening

Intronic artificial miRNAs directed against PLN were cloned into the pSM155 vector^50^, kindly provided my Michael A. Frohman (Stony Brook University, NY), using asymmetric BsmBI restriction sites (Supplementary Fig. 7). For amiRNA candidates screening, a reporter construct was obtained by inserting the full-length human PLN complementary DNA (cDNA; amplified by PCR from human heart cDNA) downstream of the luciferase coding sequence of the pGL3-control plasmid (Promega). For the PLN-R14del reporter vector, a pair of complementary primers encoding mutant PLN was annealed and inserted in the same position of pGL3-control vector. HEK-293T cells were co-transfected with both pSM155-amiRNA and reporter vectors in a 5:1 ratio using calcium phosphate transfection, and luciferase activity from cell lysates was measured 24 h post transfection using Luciferase assay reagent (Promega). Transfection efficiency was normalized by measuring the green fluorescent protein fluorescence from pSM155 vectors. PSM155 vectors containing a scrambled miRNA or a luciferase-directed miRNA were used as negative and positive controls, respectively.

### Immunocytochemistry

iPSC were cultured on matrigel-coated coverslips, fixed in paraformaldehyde and permeabilized in blocking/permeabilization buffer (2% BSA/2% FBS/0.05% NP40 in PBS) for 45 min and incubated with primary antibodies overnight at 4 °C. Then the cells were washed in PBS and incubated with Alexa-conjugated secondary antibodies (Invitrogen) diluted in blocking/permeabilization buffer (1:750). Finally, after washing in PBS the cells were counterstained with 4,6-diamidino-2-phenylindole (DAPI). Immunofluorescence images were acquired using an Olympus X41 microscope. The following antibodies were used: mouse monoclonal anti-OCT4 (1:100, Santa Cruz), goat polyclonal anti-NANOG (1:100, R&D systems), mouse monoclonal anti-SOX2 (1:100, R&D systems) and mouse monoclonal anti-SSEA-4 (1:100, R&D systems). Similarly, iPSC-derived CMs were dissociated and cultured on matrigel-coated coverslips for 4–5 days, fixed in paraformaldehyde and permeabilized in blocking/permeabilization buffer for 45 min. The cells were incubated with Alexa-conjugated primary antibodies overnight at 4 °C, washed in PBS and counterstained with DAPI. Confocal imaging was performed using a Leica SP5 confocal system. The following antibodies were used: mouse monoclonal anti-cardiac troponin T (1:200, Thermo Fisher Scientific) and mouse monoclonal anti-PLN (1:200, Bradilla).

### Immunohistochemistry

Myocardial tissues were obtained from the explanted hearts of patients carrying the PLN-R14del mutation (*n*=2) and patients with ischaemic cardiomyopathy (*n*=2) at the time of cardiac transplantation at the University Medical Center Utrecht (Department of Cardiology, Division Heart and Lungs, Utrecht, The Netherlands) with patients' consent and an approved protocol by the center's Institutional Review Board. Myocardial samples were derived from the left ventricular septum and posterolateral walls of PLN-R14del patients, and remote (non-infarcted) areas of ischemic cardiomyopathy patients. The explanted heart tissues were fixed in 10% neutral-buffered formalin processed by standard protocol and embedded in paraffin. Four-micron-thick tissue sections were deparaffinized in xylene and rehydrated in graded alcohols. Antigen retrieval was performed in a sodium citrate buffer (pH 6.0) in a pressure cooker. Myocardial sections were washed in PBS, incubated in blocking buffer (5% normal donkey serum in PBS) for 30 min at room temperature, followed by primary antibodies, rabbit polyclonal anti-Serca2a (1:100) and mouse monoclonal anti-PLN (1:100) at 4 °C overnight. After three washes in PBS, sections were incubated for 60 min with 1:750 secondary antibodies (anti-rabbit Alexa-555 and anti-mouse Alexa-488). Confocal imaging was performed using a Leica SP5 confocal system.

### Quantitative RT–PCR

Relative gene expression was determined using a two-step quantitative real-time PCR method. Total RNA was isolated with the RNeasy Isolation kit with on-column DNase I treatment (Qiagen) and reverse-transcribed using the cDNA Synthesis Kit (Quanta biosciences). Quantitative reverse transcription PCR (RT–PCR) was performed with the Quanta SYBR Green Supermix (Quanta biosciences) on the ABI Prism 7500 Real-Time PCR System (Applied Biosystems). Fold changes in gene expression were determined using the comparative *C*_T_ method (ΔΔCt) with normalization to the housekeeping genes *GAPDH* or *B2M*. The primer sequences used in the study are shown in Supplementary Table 2. The TaqMan hPSC Scorecard panel was used to assess pluripotency and trilineage differentiation potential according to manufacturer's instructions (Life Technologies). Data analysis was performed using the hPSC Scorecard software (Life Technologies).

### Pyrosequencing analysis

PCR templates for pyrosequencing were amplified from cDNA using PrimeSTAR GXL DNA Polymerase (Clontech) following the manufacturer's instructions. The pyrosequencing was performed using the PyroMark Q24 (Qiagen) and pyrograms were analysed using the PyroMark Q24 Software 2.0.

### SNP karyotyping

Single nucleotide polymorphism (SNP) karyotype analysis was performed on the Illumina's CytoSNP-850K genotyping microarrays, which measure ∼850,000 SNPs across the genome. All genomic DNA was isolated from iPSC clones according to the manufacturer's protocol (QIAGEN). Input genomic DNA (500 ng) was processed, hybridized to the array and scanned on an Illumina HiScan according to the manufacturer's instructions. Copy number variations (CNVs) were identified using the cnvPartition Pluginv.3.2.0 in GenomeStudio (Illumina) by assessing both the B-allele frequency and Log R ratios.

### Single-cell electrophysiology and Ca^2+^ imaging

The patch-clamp and Ca^2+^ imaging assays were performed on single CMs isolated from the differentiated cardiospheres and seeded on matrigel-coated glass coverslips or glass bottom dish. Assays were performed 5–7 days post seeding (30–50 days post differentiation).

### Patch-clamp studies

The action potentials were recorded by whole-cell patch-clamp technique using the EPC10 amplifier and Pulse/PulseFit software (HEKA, Germany) at 37 °C. The patch pipettes were prepared to have a typical resistance of 3–6 MΩ. The internal pipette solution contained 110 mM potassium aspartate, 20 mM KCl, 10 mM HEPES, 1 mM MgCl_2_, 0.1 mM ATP (disodium salt), 5 mM ATP (magnesium salt), 5 mM phosphocreatine (disodium salt) and 1 mM EGTA (pH 7.2). The external bath solution contained 140 mM NaCl, 5 mM KCl, 1 mM MgCl_2_, 10 mM D-glucose, 1 mM CaCl_2_ and 10 mM HEPES (pH 7.4).

### Ca^2+^ imaging

Ca^2+^ imaging was carried out with a spinning disk confocal microscope (PerkinElmer, USA) at 37 °C. Ca^2+^-sensitive dye, X-Rhod 1 (Invitrogen, 1.5 μM, 15 min at 37 °C), was loaded into the isolated CMs, followed by washing and subsequent 5 min incubation in the same HEPES buffered external solution as used in the patch-clamp experiment. Diastolic Ca^2+^ level were estimated as the background-corrected fluorescence intensity of X-Rhod 1 without any stimulation. Ca^2+^ transient recordings were sampled at 33 Hz. Electrically induced Ca^2+^ transients were triggered by an isolated field stimulator (40 V cm^−1^, 20-ms pulse at 0.5 Hz); caffeine-induced Ca^2+^ transients were triggered with a puff of caffeine (10 μM). Fluorescence intensity of CMs in sequences of time-lapse images recorded was converted to numerical data with Image J (NIH, US), followed by mathematical treatment with Origin (OriginLab, Northampton, MA) to obtain the Ca^2+^ transient parameters including the transient amplitude, resting Ca^2+^, maximum upstroke and decay velocity and decay constant.

### RNA-seq analysis

Cardiomyocytes derived from PLN-R41del iPSC cells were infected with AAV6-EGFP-miR-PLN or AAV6-EGFP-miR-luc as a negative control at an MOI of 10^4^. One week after infection, total RNA was extracted using Zymo columns and 2 μg were used to generate a RNA-seq sequencing library. Poly-A selection and mRNA-SEQ library preparation were performed at the Mount Sinai Genomics Core Facility. Sequencing (50 bases, paired ends) was performed using a Illumina HiSeq2500. Annotated reads were obtained using STAR and HTSeq and normalized to full library size. Scatter plots of read counts were realized using Graphpad PRISM. For the Venn diagram analysis, 41 human transcripts showing significant homology to the PLN miRNA target sequence (3′-GCTATAAGAGCCTCAACCATT-5′) were identified using BLASTn, and compared with the 381 transcripts significantly downregulated in cells expressing miR-PLN versus control cells expressing miR-Luc. A surface-proportional Venn diagram was obtained using the BioVenn application (http://www.cmbi.ru.nl/cdd/biovenn/index.php). RNA-seq data have been deposited in GEO (Gene Expression Omnibus) under accession code GSE65763.

### Exome sequencing

Genomic DNA samples were assessed for quantity by Qubit fluorometry (Life Technologies, Grand Island, NY) and for quality by the 2100 Bioanalyzer system (Agilent). Initial shearing of 0.5–1 μg genomic DNA to an average of 200–300 bp fragments was performed using the Covaris E210 focused acoustic energy system (Covaris). Whole genome libraries were prepared using the NEBNext DNA Library Prep kit according to the standard manufacturer's protocol (New England Biolabs). Illumina compatible paired-end adapters were used and the adapter-ligated DNA fragments was amplified by ligation-mediated PCR (KAPA Biosystems) using a reverse PCR primer containing a 6 nt barcode that allowed for multiple samples to be pooled and sequenced in the same run. The library was enriched for human exomic sequences using the SeqCap EZ Human Exome Library v3.0 capture system (Roche NimbleGen). The libraries were then sequenced with a 100 bp paired-end protocol on the Illumina HiSeq2500 according to the manufacturer's protocol (Illumina). Exome sequencing data have been deposited in GEO under accession code GSE65763.

### Identification of novel indels

Off-target indels were identified by computational approach^35^. Paired-end reads were aligned to the human reference genome hg19 using Bowtie-2.1.0, followed by manipulation using PicardTools-1.110 (sorting, duplicate marking, readGroup addition and indexing). The Genome Analysis ToolKit (GATK), version 3.1.1, was used for indel identification. Sample processing included local realignment via ‘RealignmentTargetCreator' and ‘IndelRealigner', base score recalibration via ‘BaseRecalibrator', variant calling across the three samples via ‘HaplotypeCaller' and variant score recalibration via ‘VariantRecalibrator' and ‘ApplyRecalibration'.

The set of identified indels was filtered in several steps. To remove indels near low-complexity regions the hg19 RepeatMasker database was downloaded from UCSC Genome Browser website. Every indel that contained a masked base pair within a distance of 10 bp or >33 masked basepairs within a distance of 50 bp was removed. None of the identified indels caused expansions or compression of long (>6 bp) homopolymer, so no indels were removed at this step. To generate a list of sample specific indels only indels with differing alternative alleles in both clones (L2GC1 and L2GC2) were kept. In addition, we removed all those indels in which the alternate allele was not covered by at least three reads. To identify indels that lie close to possible off-target TALEN binding sites, we generated a list of sequences by substituting ≤5 bp of each monomer's on-target binding site and aligned them to the reference genome hg19 using bowtie. Indels that are located within 100 bp of genomic binding positions were considered to lie next to an off-target binding site. About 16,500 TALEN off-target binding sites were located on opposite strands and separated by 10–22 bp and were considered as paired.

## Author contributions

I.K., F.S. and M.N. designed and conducted all aspects of the experiments, co-wrote and edited the manuscript. C.T. and D.S. derived the fibroblasts from the patient's biopsy. V.T. carried out the pyrosequencing experiments, SNP karyotyping and Scorecard analysis. S.R. analysed the RNA sequencing data and J.H. analysed the exome sequencing data. D.C. assisted in culturing the iPSC-derived cardiomyocytes. F.K., D.K., A.K. and D.T. were the cardiologists involved in the care of the patient and obtained the skin biopsy. N.C. assisted with the karyotyping of cell lines. J.M.I.H.G., A.V. and F.W.A. obtained cardiac tissues from patients with the phospholamban R14del mutation or ischaemic cardiomyopathy in the Netherlands. L.R., C-W.K. and R.A.L. obtained and analysed the electrophysiological data from the iPS-derived cardiomyocytes. J.C.W. and J.-S.H. contributed to the experimental design. E.K. was responsible for identifying the R14del mutation in the patient with cardiomyopathy and editing the manuscript. R.J.H. oversaw the overall project, designed the experiments, was responsible for funding, and drafted and edited the manuscript.

## Additional information

**Accession codes:** RNA and Exome sequencing data have been deposited in GEO under accession code GSE65763.

**How to cite this article:** Karakikes, I. *et al.* Correction of human phospholamban R14del mutation associated with cardiomyopathy using targeted nucleases and combination therapy. *Nat. Commun.* 6:6955 doi: 10.1038/ncomms7955 (2015).

## Supplementary Material

Supplementary Information Supplementary Figures 1-9, Supplementary Tables 1-2.

Supplementary Movie 1 Parasternal long axis view shows enlarged left ventricle, impaired left ventricular contraction and enlarged left atrium. Left ventricular end-diastolic diameter: 52 mm, Left ventricular systolic diameter: 39mm. Estimated left ventricular ejection fraction: 47%.

Supplementary Movie 2 Spontaneous beating activity of the iPSC-R14del derived cardiomyocytes at 15 days post differentiation.

## Figures and Tables

**Figure 1:**
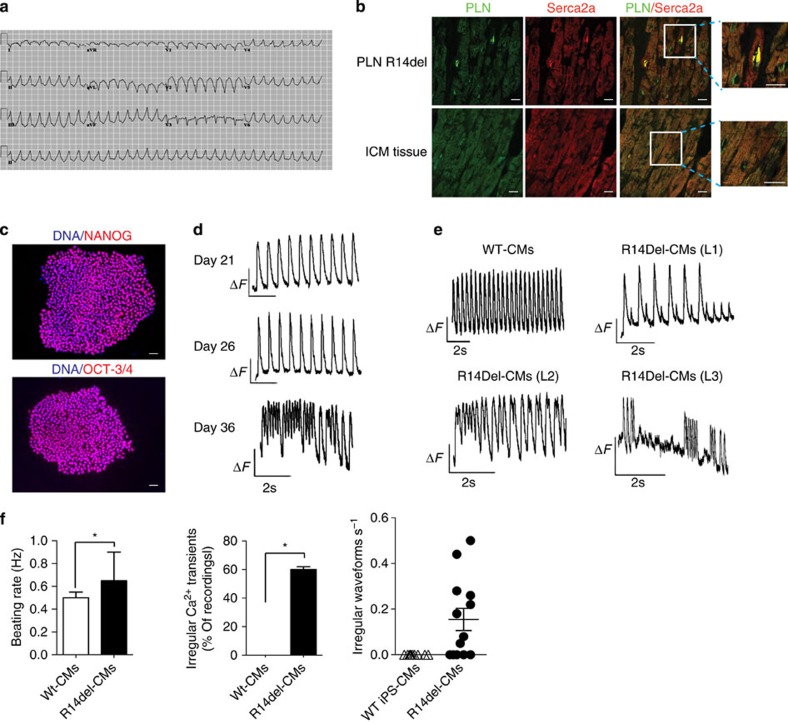
Modelling of DCM using patient-specific iPSCs carrying a R14del mutation in *PLN*. (**a**) Twelve lead electrocardiogram from a patient showing wide complex ventricular arrhythmias; (**b**) Representative immunohistochemistry images of PLN protein expression in explanted heart tissue from DCM patients carrying the R14del mutation and patients diagnosed with ischaemic cardiomyopathy (ICM). Scale bar, 25 μm; (**c**) Immunostaining for the pluripotent markers OCT-3/4 and NANOG in R14del patient-specific iPSCs. Nucleus was counterstained with DAPI. Scale bar, 10 μm; (**d**) Representative Ca^2+^ transients of R14del-CMs during the course of differentiation; (**e**) Representative Ca^2+^ transients of control and R14del-CMs derived from three independent iPSC clones at 36 days post differentiation, s=seconds. (**f**) Quantification of percentages of control and R14del-CMs exhibiting irregular Ca^2+^ transients at day 36 of differentiation. The beating rate was measured as number of events (peaks) per time (sec) during a stimulation of 0.5 Hz. Irregular Ca^2+^ transients were determined by quantifying the percentage of beating areas exhibiting irregular Ca^2+^ transient. The number of irregular peaks were counted during each recording for 1 min at 0.5 Hz. Values represent mean±s.d. (*n*=13 of R14del-CMs derived from two independent iPSC clones L2 and L3). *P<0.05 (unpaired student's *t*-test).

**Figure 2:**
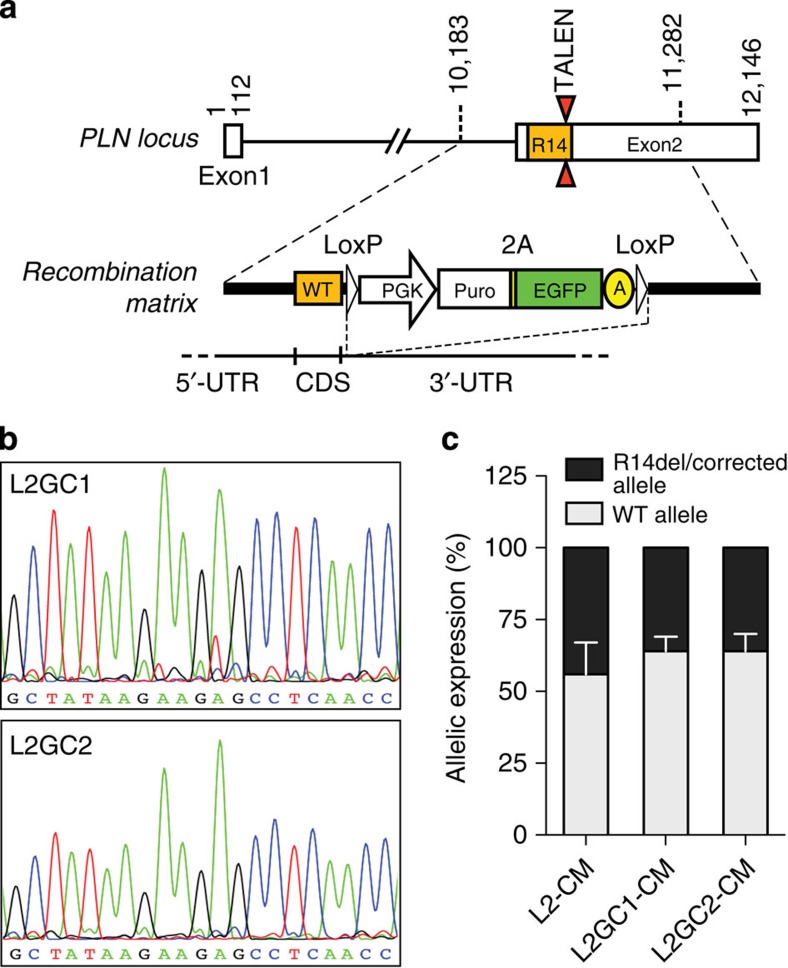
TALEN-mediated correction of R14del mutation. (**a**) Design of the exchange matrix to correct the R14del. (**b**) Confirmation by Sanger sequencing of the correction of the R14del mutation in two individual iPSCs clones derived from the L2 clone. (**c**) Pyrosequencing analysis of the PLN allelic expression in CMs derived from the parental and TALEN-corrected clones. Data represent mean±s.d. of three independent cardiomyocyte differentiations.

**Figure 3:**
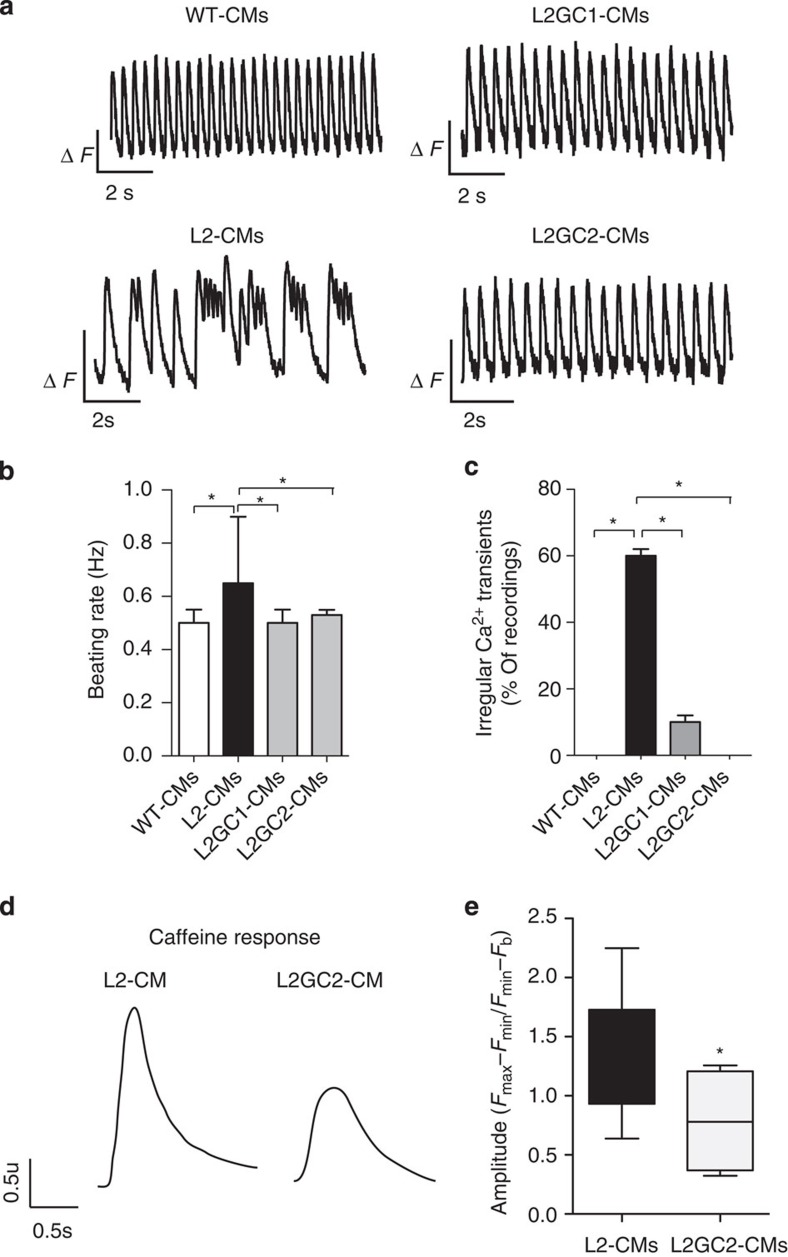
Gene correction ameliorates Ca^2+^ abnormalities. (**a**) Representative Ca^2+^ transients of cardiomyocytes derived from WT-, L2- and isogenic TALEN-corrected (L2GC1 and L2GC2) clones at 36 days post differentiation. (**b**) Beating frequency. (**c**) Quantification of percentages of isogenic CMs exhibiting irregular Ca^2+^ transients at day 36 of differentiation. The beating rate was measured as number of events (peaks) per time (sec) during a stimulation of 0.5 Hz. Irregular Ca^2+^ transients were determined by quantifying the percentage of beating areas exhibiting irregular Ca^2+^ transient. The number of irregular peaks were determined during each recording for 1 min at 0.5 Hz. Mean±s.d. (*n*=13 recordings of L2-CMs; *n*=16 recordings of L2GC1; *n*=40 recordings of L2GC1); *P<0.05 (ANOVA, Tukey's Multiple Comparison Test). (**d**) Representative caffeine-induced Ca^2+^ transients in single cells; (**e**) Amplitude analysis of caffeine-induced Ca^2+^ transients in single cardiomyocytes. A caffeine puff (10 mmol l^−1^) was applied and caffeine-induced amplitude was assessed. Values represent mean±s.e.m. (*n*=15 L2-CM and *n*=4 L2GC2-CM). *P<0.05 (unpaired student's *t*-test).

**Figure 4:**
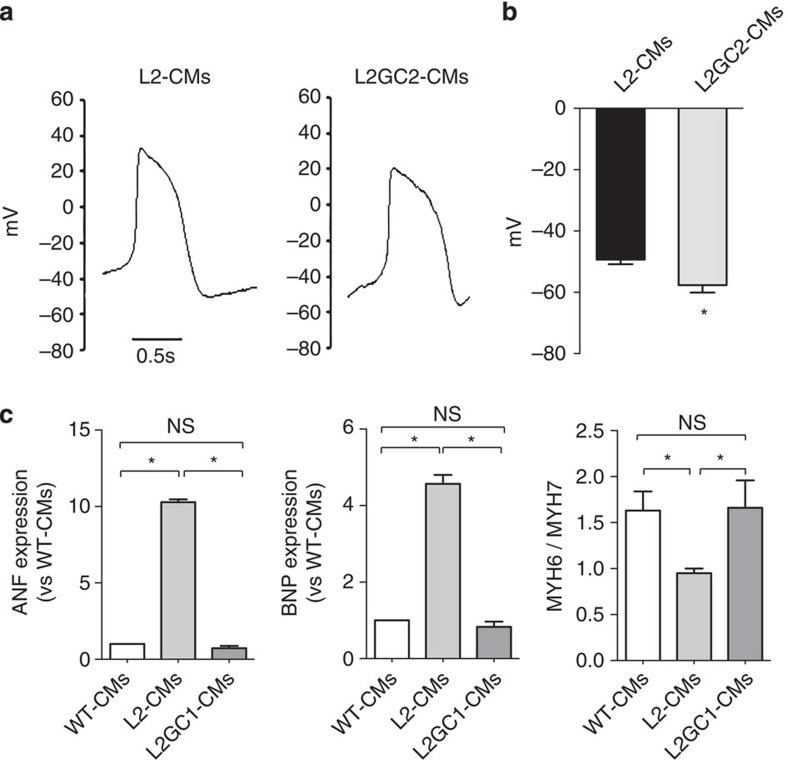
Gene correction improves elephysiological and hypertrophic phenotypes. (**a**) Representative action potentia (AP) waveforms of spontaneously beating cardiomyocytes. The AP properties of single cells were analysed using the patch-clamp method; (**b**) Analysis of maximum diastolic potential. Values represent mean±s.e.m. (*n*=14 L2-CM and *n*=22 L2GC2-CM). *P<0.05 L2-CMs versus L2GC2-CMs (unpaired student's *t*-test); (**c**) Quantitative PCR analysis of gene expression of cardiac hypertrophy markers ANF, BNP, MYH6 and MYH7 in isogenic CMs. ANF and BNP gene expression was normalized to GAPDH housekeeping gene and expressed as relative expression to the WT-CMs. MYH6 and MYH7 expression was normalized to GAPDH housekeeping gene and presented as MYH6/MYH7 ratio. Values represent mean±s.e.m. (*n*=6 per group). *P<0.05 (unpaired student's *t*-test). NS, not significant.

**Figure 5:**
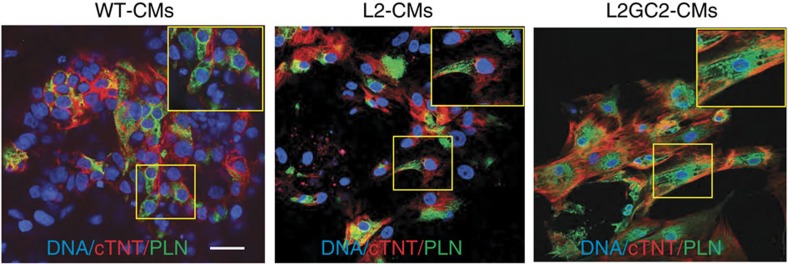
PLN protein distribution in cardiomyocytes. Representative immunofluorescence images showing the intracellular protein distribution of phospholamban (PLN) and cardiac troponin T (cTNT) in WT-, L2- and L2GC2-CMs. Nuclei were counterstained with DAPI. Scale bar, 25 μm.

**Figure 6:**
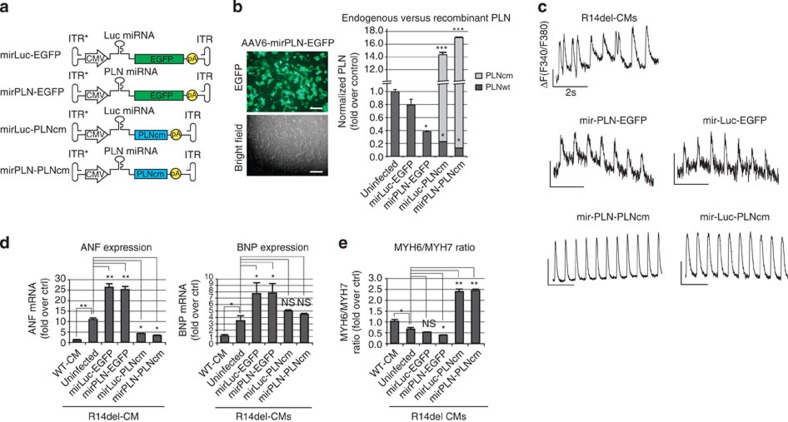
AAV-mediated gene therapy. (**a**) Map of AAV vectors. The asterisk denotes the ITR mutation necessary for self-complementary genome packaging; (**b**) Transduction of iPS-CMs by self-complementary AAV vectors pseudotyped in AAV6 capsids. CMs derived from R14del-iPSC cells were infected at an MOI of 4 × 10^4^ viral genomes per cell on day 28 post differentiation. Transduction efficiency was measured 7 days post infection by green fluorescent protein fluorescence (left; scale bar,100 μm) or qRT–PCR with primers specific for the endogenous and viral PLN (PLNwt and PLNcm, respectively). Values are normalized to uninfected R14del-CMs; (**c**) Representative traces of intracellular Ca^2+^ transient waves of electrically stimulated (0.5 Hz) R14del-CMs performed 7 days post infection with the indicated AAV6 constructs; (**d**) Expression of cardiac hypertrophy markers ANF and BNP measured by qRT–PCR and normalized to GAPDH expression. Values are expressed as relative expression to WT-CMs; (**e**) Gene expression analysis of the myosin heavy chain isoforms ratio, MYH6/MYH7, measured by qRT–PCR. Values are normalized to WT-CMs. (**b**,**d**,**e**) Values represent the mean+s.d. (*n*=3). *P<0.05, **P<0.01, ***P<0.001, NS=*P*>0.05 (unpaired student's *t*-test). NS, not significant.

**Table 1:** Action potential parameters in mutated (L2) and isogenic (L2-GC2) CMs.

**Parameters**	**L2-CM*****n*****=14**	**L2-GC2-CM*****n*****=22**
Frequency (Hz)	0.66±0.07	0.55±0.05
APA (mV)	59.30±3.70	58.20±2.20
Upstroke velocity (mV ms^−1^)	3.56±0.54	1.97±0.24*
Decay velocity (mV ms^−1^)	−0.33±0.04	−0.29±0.04
APD90 (ms)	543±74	710±69
MDP (mV)	−49.3±1.50	−57.70±2.40^#^

APA, action potential amplitude; APD90, AP duration measured at 90% repolarization; CM, cardiomyocyte; MDP, maximum diastolic potential; iPSC, induced pluripotent stem cells; TALEN, transcription activator-like effector nucleases.

Data are represented as mean±s.e.m. Two-tailed Student's *t*-test was used to test the mean between groups, *P<0.05, ^#^*P*<0.01.
