# Supplementary material for: Correction of human phospholamban R14del mutation associated with cardiomyopathy using targeted nucleases and combination therapy
Source: Nat Commun. 2015 Apr 29;6:6955. doi: 10.1038/ncomms7955 (PMC4421839; doi:10.1038/ncomms7955)
Supplement: Supplementary Information — Supplementary Figures 1-9, Supplementary Tables 1-2. [file ncomms7955-s1.pdf]

Supplementary Figure 1

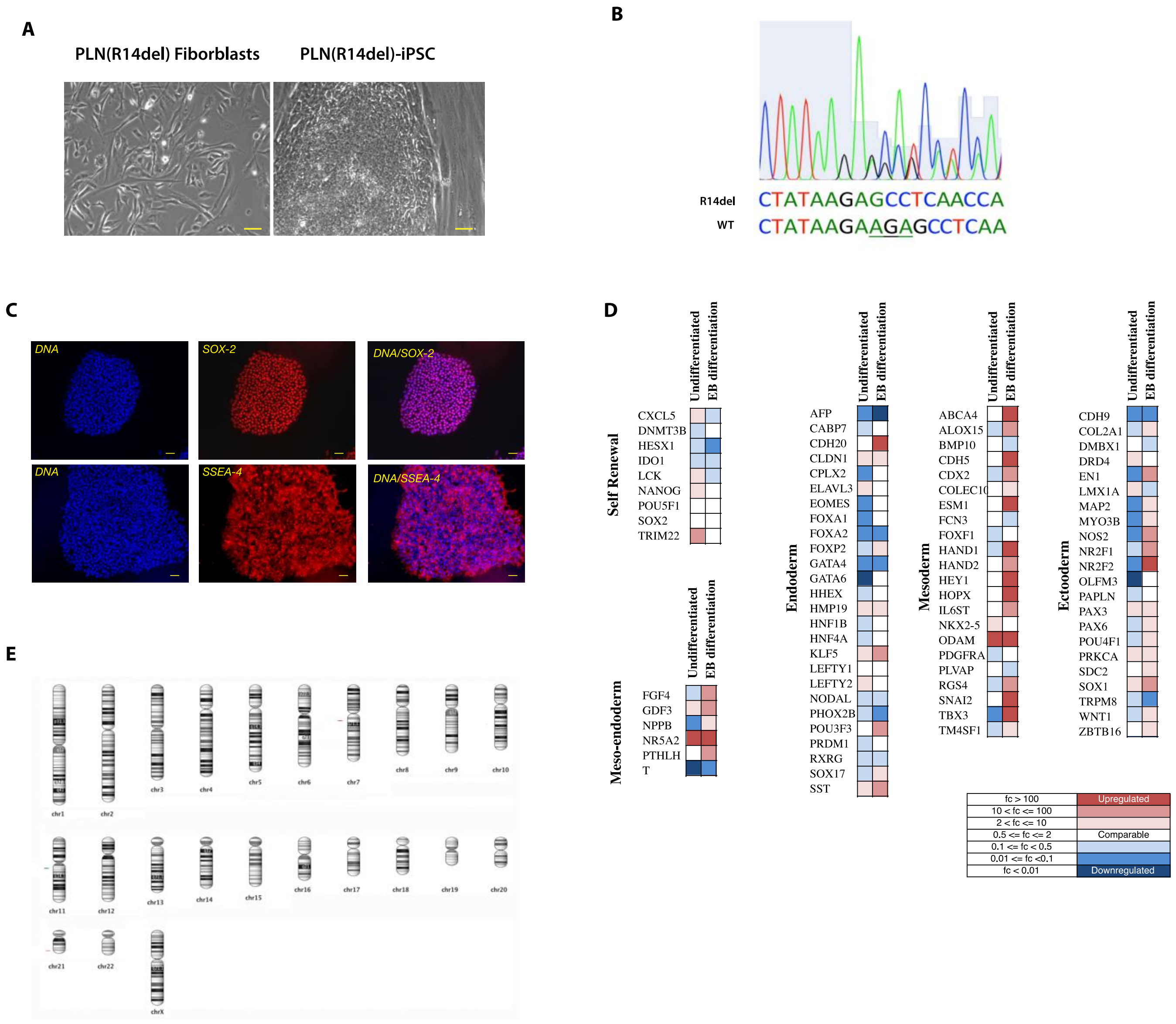

Supplementary Figure 1. Derivation and characterization of iPSC derived from a patient harboring a heterozygous R14del mutation in the *PLN* gene. **A**) Phase contrast images of the dermal fibroblasts and a representative iPSC colony derived from a patient carrying the R14del. The fibroblasts were reprogrammed to iPSC with a reprogramming cocktail of five human synthetic mRNA reprogramming factors encoding Oct4, Sox2, c-Myc, Klf4, and Lin28. **B**) Confirmation of the R14del heterogeneous mutation in the *PLN* coding region in dermal fibroblast-derived iPSC cells by Sanger sequencing analysis. One of the alleles shows the deletion of three nucleic acids, AGA, corresponding to the amino acid Arginine at position 14 (R14del), while the other allele is unaffected (WT). **C**) Representative immunofluorescence images of patient-specific iPSC-R14del colonies stained for the pluripotency-associated markers SOX-2 and SSEA-4. Scale bar = 50μm**. D)** Gene expression plots of the TaqMan® hPSC Scorecard™ Panel. Colors correlate to the fold change in gene expression of the indicated genes relative to the undifferentiated reference set. **E)** Karyotype analysis.

Supplementary Figure 2

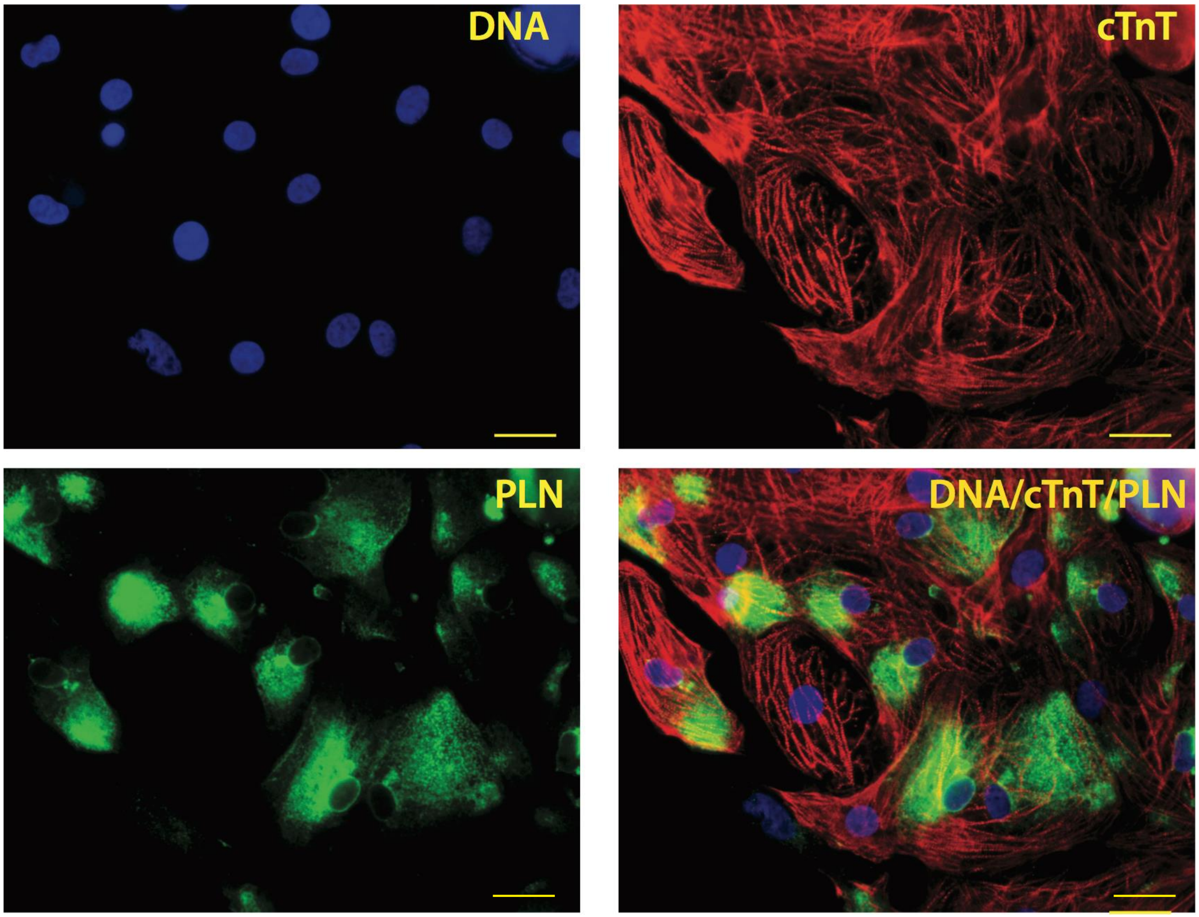

Supplementary Figure 2. Directed differentiation of patient-specific iPSCs into cardiomyocytes. Representative immunofluorescence images of iPSC-derived cardiomyocytes stained for the cardiomyocyte-specific marker *cTnT* (red) and PLN (green). DNA was counterstained with DAPI (blue). Three individual iPSC-R14del lines (L1, L2, L3) were differentiated. Scale bar = 20µm. cTnT: cardiac troponin T.

Supplementary Figure 3

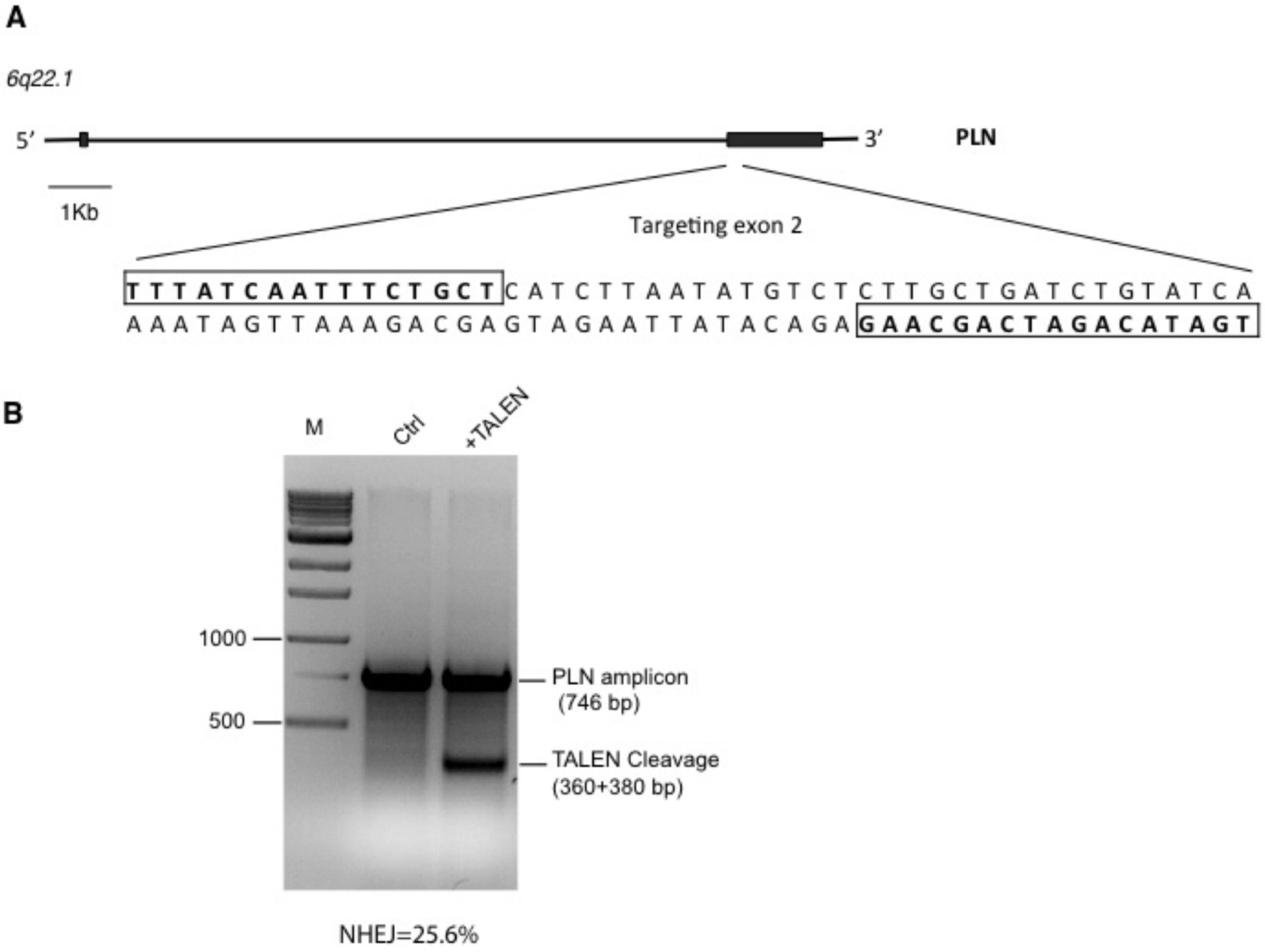

Supplementary Figure 3. Validation of TALEN-mediated editing of the *PLN* locus. A) This schematic shows a pair of TALENs designed to target the *PLN* locus in the human genome. The left and right TALENs recognize the top and bottom strands of the target sites separated by a 16-bp spacer, and each recognition site begins with a T. The nucleotide sequences of the target sites are shown. Each TALE DNA-binding domain is fused to the catalytic domain of FokI endonuclease, which dimerizes and cleaves the targeted DNA in the spacer region upon binding. **B)** Estimation of cleavage efficiency of the *PLN* TALEN pair. Agarose gel showing the Surveyor nuclease result from the *PLN* TALEN pair. DNA was prepared from HEK293 cells transfected with left and right TALENs for 72h. The TALEN cleavage efficiency is calculated based on the fraction of cleaved DNA by the Surveyor nuclease, which recognizes and cuts the mismatch in DNA hybrids. The two lower bands indicated by the arrows are Surveyor-cleaved DNA products. The expected sizes of the digested fragments and the estimated modification efficiency (% NHEJ) are shown. NHEJ: Non-homologous end joining.

Supplementary Figure 4

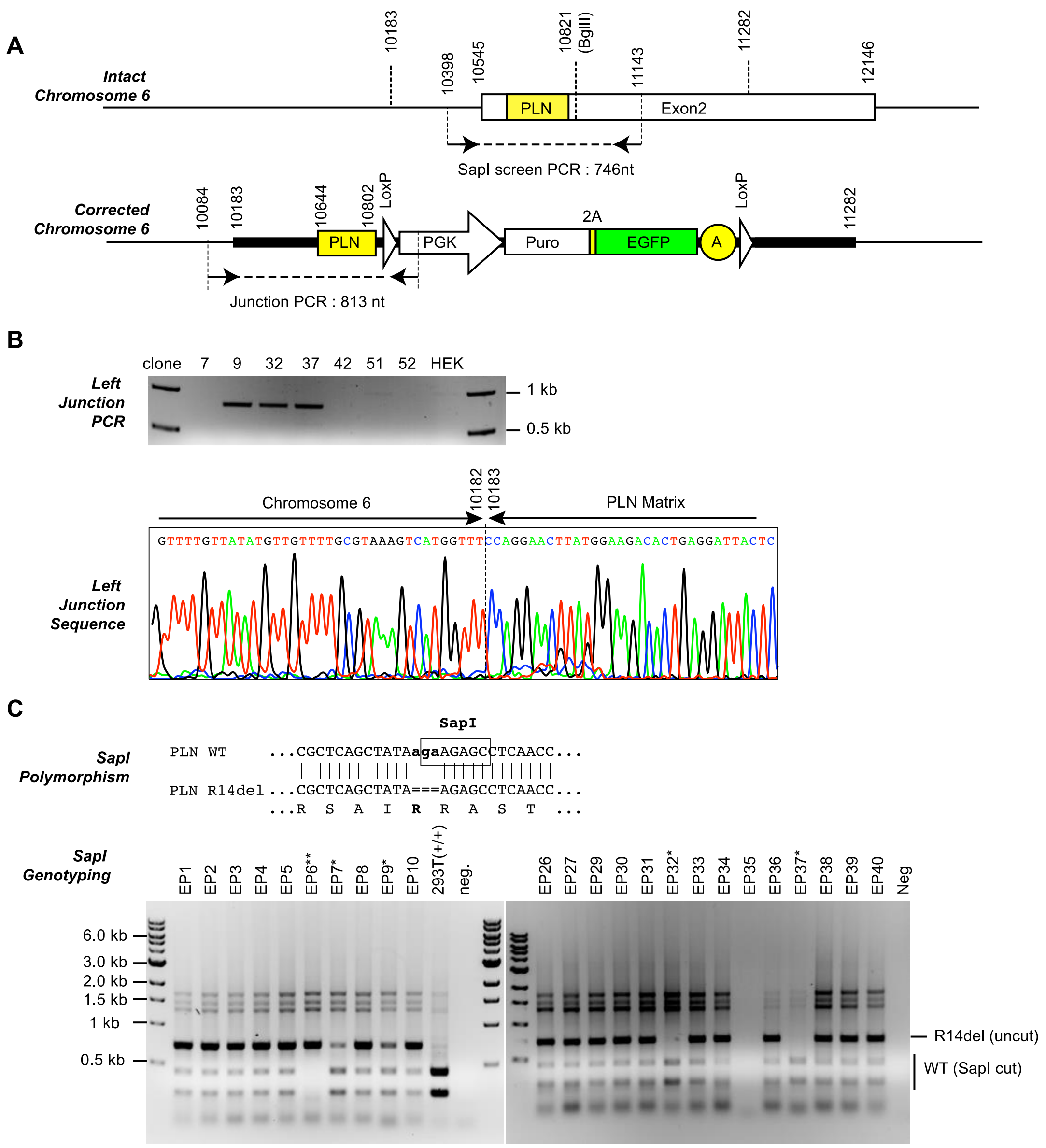

Supplementary Figure 4. Design and screening of PLN gene correction by TALEN-enhanced recombination. A) Structure of phospholamban gene before and after TALEN-mediated recombination. Numbers refer to the nucleotide positions of human phospholamban genomic sequence. The BglII restriction site used to insert the selection cassette is represented. Yellow box: phospholamban coding sequence. Arrows indicate the positions of the primers used for PLN allelic discrimination (upper panel) or for junction screen (lower panel). **B)** Junction PCR screen and sequencing. Genomic DNA from puromycin-resistant iPS clones was used as a template for PCR with a forward primer specific for the chromosome 6 sequence preceding the homologous recombination arm and a reverse primer specific for the PGK promoter, yielding a 813-nt fragment (upper panel). Lower panel: sanger sequencing of purified PCR fragment showing the junction between chromosome 6 and the left homologous recombination arm. **C)** Restriction fragment polymorphism analysis of the non-corrected PLN allele. A 746-nt fragment containing the entire PLN coding sequence was amplified by PCR from the intact (non-corrected) chromosome and digested with SapI endonuclease, which cuts only the wild-type PLN allele. Digestion products were run on 2% agarose gels.

Supplementary Figure 5

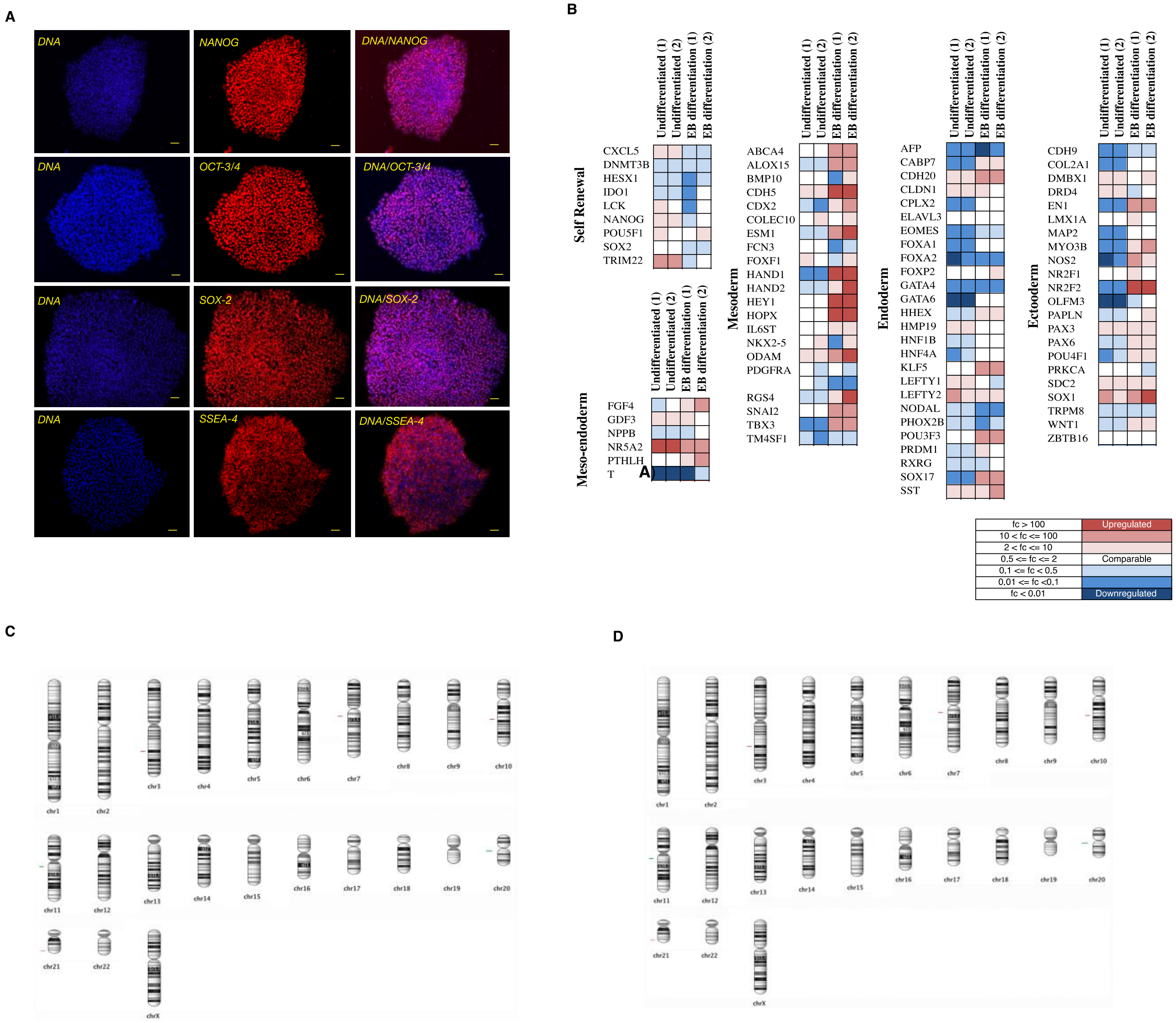

Supplementary Figure 5. Characterization of TALEN-mediated gene corrected iPSCs. A) Representative immunofluorescence images of gene corrected iPSC colonies stained for the pluripotency-associated markers OCT-3/4, NANOG, SOX-2 and SSEA-4. Scale bar = 50µm. **B)** Gene expression plots of the TaqMan® hPSC Scorecard™ Panel. Colors correlate to the fold change in expression of the indicated genes relative to the undifferentiated reference set. **C-D)** Karyotype analysis of TALEN-corrected clones, L2GC1 and L2GC2, respectively.

Supplementary Figure 6

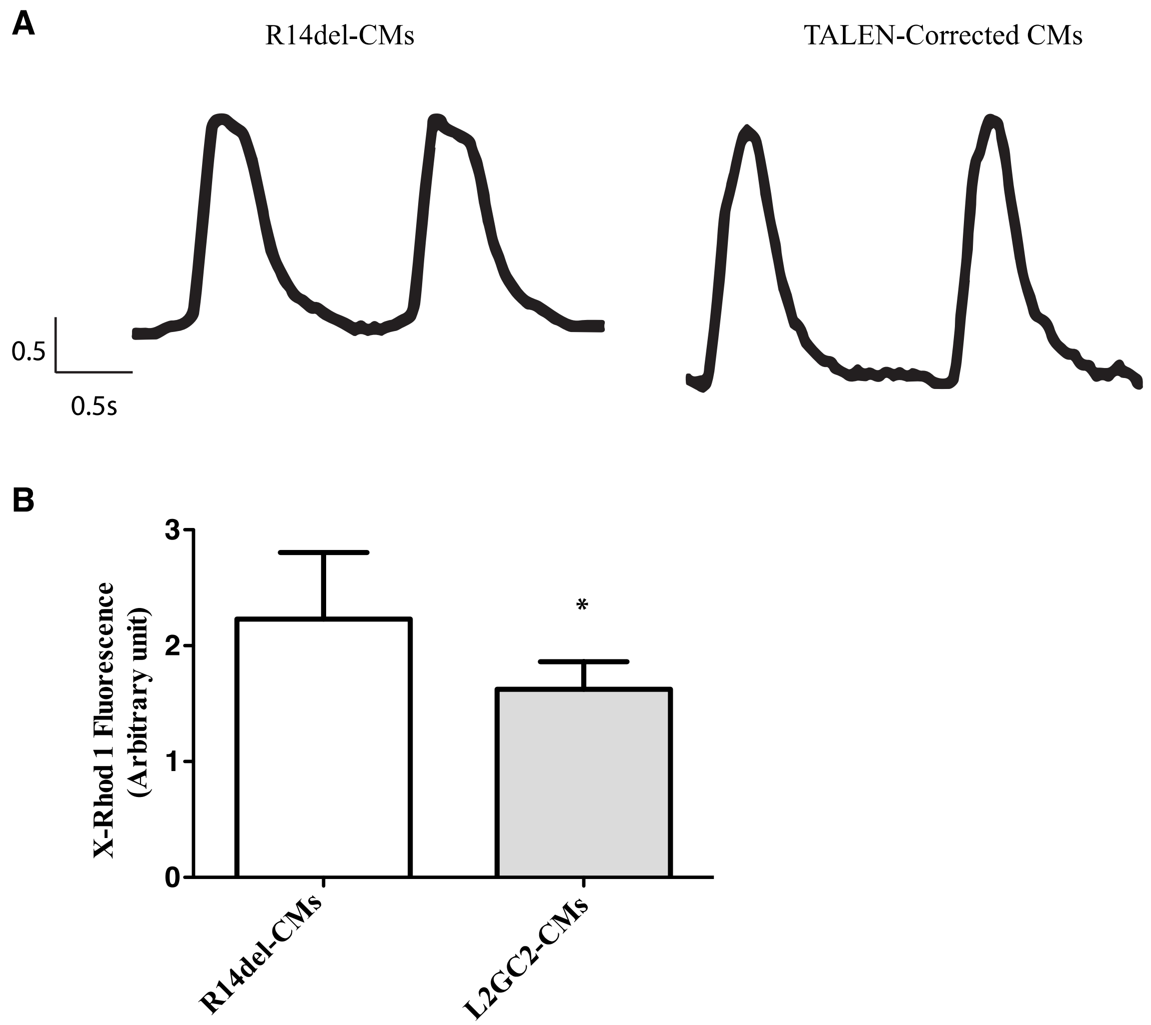

Supplementary Figure 6. Single cell Ca^{2+} imaging analysis. Single CMs Ca^{2+} imaging assay in the PLB mutant (R14del) and TALEN-mediated corrected (L2GC) cardiomyocytes (CMs). **A)** Representative Ca^{2} transients. **B**) Diastolic Ca^{2+} levels were estimated as the background-corrected fluorescence intensity of X-Rhod 1 in R14del-CMs (n=27) and L2GC-CMs (n=4) at rest. The data presented are Mean ± SD. *P > 0.05 (unpaired student's t-test).

Supplementary Figure 7

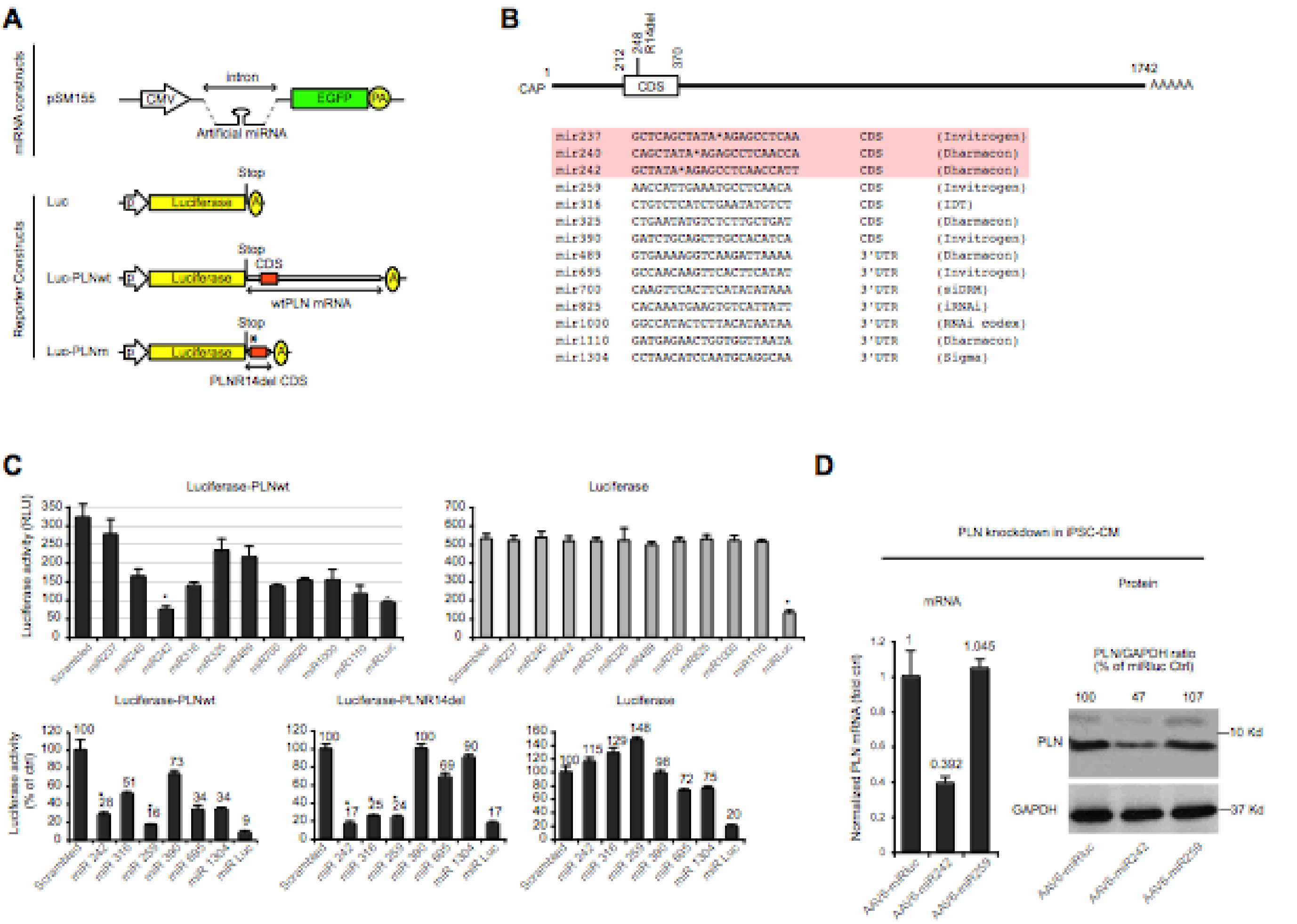

Supplementary Figure 7. Suppression of PLN expression by AAV-mediated RNAi. A) Schematic representation of the intronic miRNA expression vector pSM155 (upper panel) and luciferase reporter vectors (lower panel). PLN miRNA reporter vectors were obtained by inserting the full-length PLN cDNA (for wild-type PLN) or the PLN-R14del coding sequence downstream of the firefly gene in the pGL3-ctrl plasmid. **B)** Mapping of candidate miRNAs along the phospholamban transcript. Artificial miRNA candidates specific for the PLN-R14del are highlighted. Asterisks indicate the position of R14 (AGA codon) deletion. The numbering of miRNAs reflects the first nucleotide position on the PLN transcript. **C)** Representative PLN miRNA screening with luciferase reporter constructs. HEK-293T cells were cotransfected with pSM155-miRNA vectors and reporter vectors, and luciferase activity of lysates was quantified. Upper panels: values represent the raw luciferase activity (RLU) normalized to GFP fluorescence (arbitrary units) to account for differences in transfection efficiency. Lower panels: GFP-normalized luciferase activity was expressed as the percentage of scrambled miRNA control. Values represent the mean + SD of triplicate experiments. *P<0.05 (unpaired student's t-test). **D)** Knock-down of endogenous PLN in normal IPS-CMs following infection with AAV6-miRNA. Cells were infected with AAV6 encoding luciferase miRNA, mir242 or mir259 at an MOI of 10^4 vg/cell, and PLN expression was measured after 7 days by qRT-PCR (left panel) or western blot (right panel). Values indicate the ratio of PLN expression normalized to cells infected with AAV6-mirLuc control.

Supplementary Figure 8

A B C

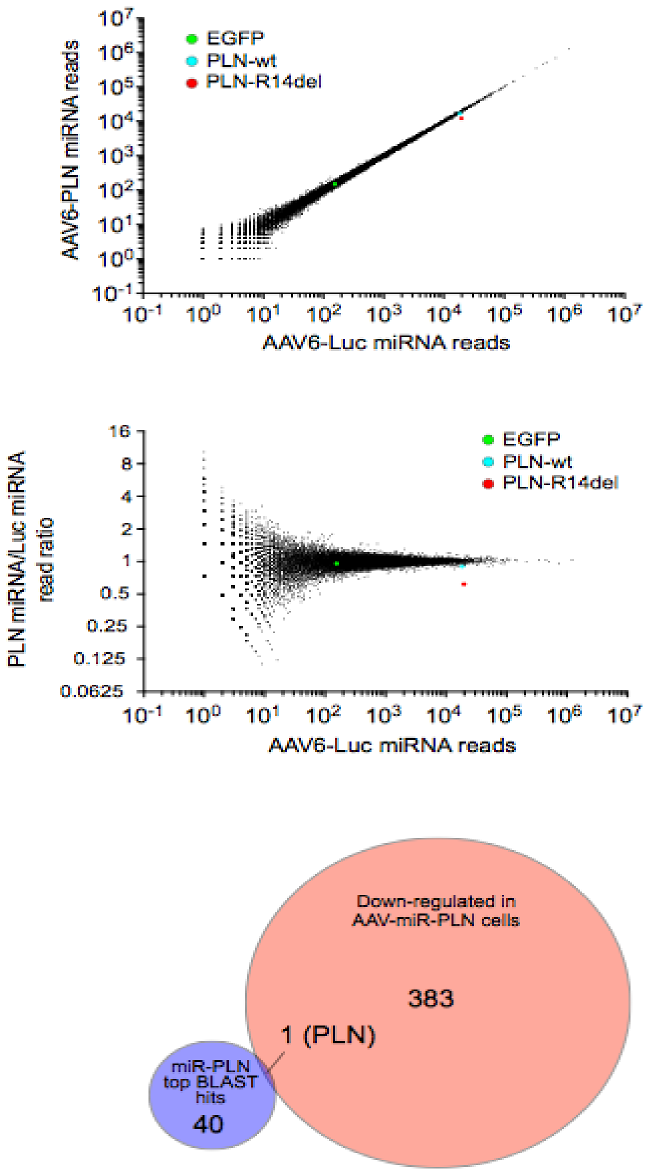

Supplementary Figure 8. confirmation of PLN microRNA specificity by RNA-seq analysis.
A) Transcriptome analysis of PLN R14del iPSC-CMs infected by AAV6-EGFP-PLN miRNA versus AAV6-EGFP-luc miRNA. The scatter plot represents the absolute RNA-seq read counts. Plots corresponding to the wild-type PLN allele (blue), the R14del PLN allele (red) and EGFP (green) are indicated. **B)** Scatter plot representing mRNA variability (Y axis) as a function of absolute counts (X axis). Note that transcript expression variability is increased among low-count occurrences (<100 counts) suggesting intrinsic, non-significant variations due to the library generation or sequencing process. **C)** Proportional Venn diagram showing the degree of overlap between the 383 transcripts significantly down-regulated in PLN miRNA-treated cells and the 41 human sequences most similar to the PLN miRNA target sequence.

Supplementary Figure 9

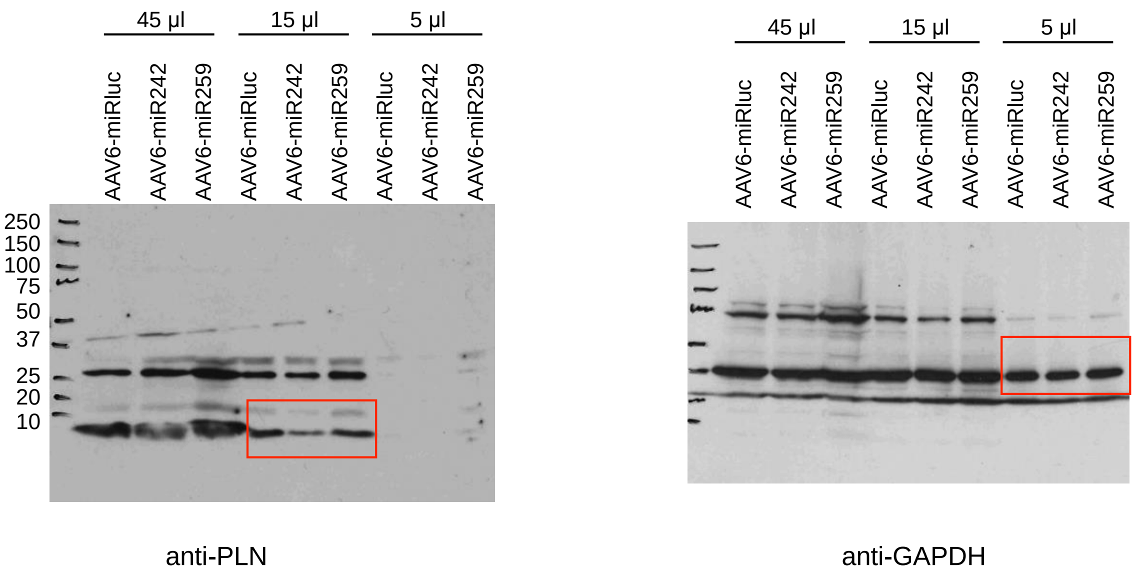

Supplementary Figure 9. The un-cropped images of the western blots showed in Supplementary Figure 8D. Boxes indicate the cropped regions.

Supplementary Table 1

		L2GC1	L2GC2	L2
**Raw indel calls**	Total	125095	168972	154233
	Missed	62355	33032	
	De-novo	33217	47771	
**Post low complexity filter**	Total	63541	82595	76388
	Missed	28399	15599	
	De-novo	15552	21806	
**Post homopolymeric filter**	Total	63541	82595	76388
	Missed	28399	15599	
	De-novo	15552	21806	
**Clone specific indels**	Total	12024	31078	33391
	Missed	24344	11544	
	De-novo	2977	9231	
**Clone specific indels, >2 reads**	Total	3211	12551	9871
	Missed	7891	2242	
	De-novo	1231	4922	
**Indels next to off target TALENS**	Total	850	3367	2631
	Missed	2099	595	
	De-novo	318	1331	
**Indels between pair of off target TALENS**	Total	3	5	4
	Missed	4	0	
	De-novo	3	1	

Supplementary Table 1. Paired-end reads were aligned to the human reference genome, followed by the identification of indels. The table shows the number of identified indels in the clones L2GC1 and L2GC2 as well as in the parental cell line (L2). The column missed labels those indels that were characterized in the parental cell line, but not in the corresponding clone, while de-novo labels indels that are found in the individual clone, but not in the parental cell line. Filtering steps were done as described in supplementary methods. Only 318 de-novo indels in L2GC1 and 1331 indels in L2GC2 are close to possible off-target TALEN binding sites, while only 3 de-novo indels in clone L2GC1 and 1 in clone L2GC2 lie between a pair of off-target TALEN binding sites.

Supplementary Table 2

Gene name	Forward primer	Reverse primer
**PLNwt**	5’-CTCACTCGCTCAGCTATAAGAAG	5’-AGAGAAGCATCACGATGATACAG
**PLNcm**	5’-GTGCAGTACCTGACCAGAAG	5’-CAGCAAGCAGATCAGAATCAAG
**ANF**	5’-CACAGATCTGATGGATTTCAAGA	5’-CCTCATCTTCTACCGGCATC
**BNP**	5’-AAGGGTCTGGCTGCTTTG	5’-CAGCCAGGACTTCCTCTTAATG
**MYH6**	5’-GGAAGACAAGGTCAACAGCCTG	5’-TCCAGTTTCCGCTTTGCTCGCT
**MYH7**	5’-GGAGTTCACACGCCTCAAAGAG	5’-TCCTCAGCATCTGCCAGGTTGT
**Beta2M**	5’-GGGATCGAGACATGTAAGCAG	5’-CAAGCAAGCAGAATTTGGAA
**GAPDH**	5’-GGAAGGTGAAGGTCGGAGTC	5’-GGAAGATGGTGATGGGATTTC

Supplementary Table 2. qPCR Primer Sequences used in the study.
